# Circulating progenitor cells in hypertensive subjects: Effectiveness of a treatment with olmesartan in improving cell number and miR profile in addition to expected pharmacological effects

**DOI:** 10.1371/journal.pone.0173030

**Published:** 2017-03-16

**Authors:** Giuseppe Mandraffino, Caterina Oriana Aragona, Valentina Cairo, Michele Scuruchi, Alberto Lo Gullo, Angela D’Ascola, Angela Alibrandi, Saverio Loddo, Sebastiano Quartuccio, Carmela Morace, Enricomaria Mormina, Giorgio Basile, Antonino Saitta, Egidio Imbalzano

**Affiliations:** 1 Department of Clinical and Experimental Medicine, University of Messina, Messina, Italy; 2 Department of Biochemical, Physiological and Nutritional Sciences, University of Messina, Messina, Italy; 3 Department of Statistics, University of Messina, Messina, Italy; 4 Department of Biomedical Sciences and of Morphologic and Functional Images, University of Messina, Messina, Italy; George Washington University School of Medicine and Health Sciences, UNITED STATES

## Abstract

CD34+ circulating progenitor cells (CD34+CPCs) are a population of multipotent cells which can delay the development of atherosclerosis and cardiovascular disease (CVD) in conditions of increased CV risk. MicroRNAs (miRs) 221 and 222 modulate different genes regulating angiogenesis and inflammation; moreover, miR221/22 have beenshown to participate in differentiation and proliferation of CD34+CPCs, inhibiting cell migration and homing. miR221/222 in CD34+CPCs from hypertensive subjects are also increased and associated with CD34+cell number and reactive oxygen species (ROS). We evaluated CD34+CPC number, intracellular miR221/222 and ROS levels, arterial stiffness (AS)and echocardiography indices at baseline (T0).Then, after a six-month treatment with olmesartan, 20 mg/day (T1), in 57 hypertensive patients with left ventricular hypertrophy (LVH) and with no additional risk factor for CVD, and in 29 healthy controls (baseline),fibrinogen, C-reactive protein (CRP), glucose and lipid profiles were also evaluated.At T1, blood pressure values, CRP and fibrinogen levels, ROS and miR221/222 were significantly decreased (all p <0.001), as were AS indices and LV mass index (p<0.001), while cell number was increased (p<0.001). Olmesartan is effective in reducing miR and ROS levels in CD34+CPCs from hypertensive subjects, as well as in increasing CD34+CPC number, providing multilevel CV protection, in addition to its expected pharmacological effects.

## Introduction

Circulating progenitor cells (CD34+CPCs), including a cell subset defined as endothelial progenitor cells (EPCs),are recognised to contribute to postnatal vasculogenesis and to endothelial homeostasis,delaying the development of atherosclerosis and cardiovascular disease (CVD)[1]. A broad range of cell types of different organs and systems, including cardiomyocytes, smooth muscle cells, and EPCs, as well as hematopoietic, stromal, and epithelial cells, may derive from CD34+CPCs; however, itis currently unclear how CD34+CPCs may differentiate into mature cells of specific lineages[1,2,3,4]. It has been suggested that circulating cells expressing the surface antigen CD34 may share both hematopoietic and angiogenic properties[1,5,6,7]; accordingly,CD34+ cell count has been questioned as a marker of regenerative/reparative potential, and the findings appear to be encouraging[1,2,6,8,9].

MicroRNAs (miRs) are small non-coding ribonucleic acid molecules regulating gene expression at the post-transcriptional level[10,11,12].miRs play a pivotal role in modulating several pathways of physiological relevance, such as endothelial lineage differentiation[13], vascular homeostasis[14,15,16,17], and blood pressure (BP)[18,19,20,21,22]. Alterations in miR expression profiles have been seen to associate with impaired cellular function and disease development[23], including CVD[24,25].

miR-221 and miR-222 (miR221/222) have been identified in CD34+ cells[13]. The pathways and molecules regulating miR221/222 expression in human progenitor cells are not known. It has been reported that miR221/222affect cell migration and proliferation by reducing the expression of c-kit and of the receptor for stem cell factor[13], and, indirectly, by inhibiting endothelial nitric oxide (NO) synthase expression[26]. Moreover, the over-expression of miR221/222 may promote apoptosis[13],and induce the production of inflammatory molecules in endothelial cells[27]. miR221/222are also suggested to be critically involved in vascular homeostasis and angiogenesis[13,15,16,26].

In recent studies, we investigated the number and function of CD34+ cells in subjects with different cardiovascular (CV) risk factors, including ageing[28], smoking[29], rheumatoid arthritis[30]and hypertension[31]. In hypertensive patients with different degrees of CV involvement, and in particular in hypertensive patients with isolated arterial stiffening (AS) or with both carotid intima-media thickening and left ventricular hypertrophy (LVH),we evaluated the expression of miR221/222 in CD34+ cells, as well as the associations between CD34+CPC number, intracellular miR221/222,and redox balance, including reactive oxygen species (ROS) production and antioxidant enzymes[31].We found increased miR221/222expression and higher ROS levels in CD34+CPCs. However, in AS hypertensive patients, redox balance and miR expression were associated with the increasedCD34+CPC number, while in hypertensive patientswith more advanced organ involvement, particularly with LVH, the greater increases in miRs and ROS were associated with a lower CD34+CPC number. This suggests that miR221/222 expression is enhanced in CPCs from hypertensive subjects and that miRs and ROS may influence CPC number.

In the present study, we aimed to evaluate whether in hypertensive patients already diagnosed with LVH, a 6 month-treatment with olmesartan medoxomil, an angiotensin II-type1 receptor (ATR1) blocker (ARB), is effective in reducing the expression of mirR221/222 in CD34+ progenitor cells and whether such reduction is correlated with changes in BP values, CD34+CPC number and intracellular ROS levels.

Our results indicate that miR221/222 expression and ROS levels in CD34+CPCs may be regulated by ATR1 in human CD34+CPC.

## Materials and methods

### Subjects

**Fig 1:**
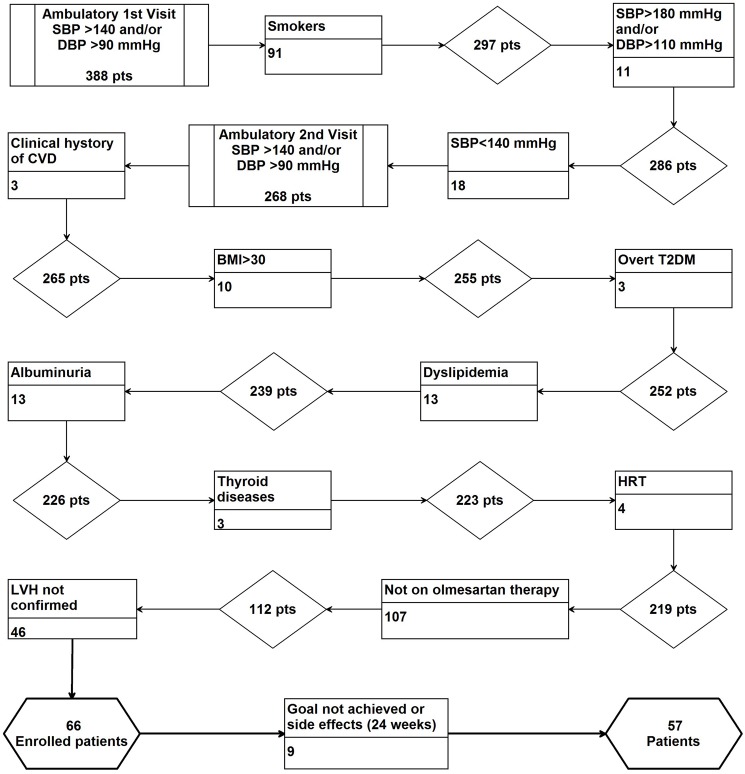
Flow diagram for patient exclusion.

The data used for this study were obtained from the medical records filed at the Hypertension Clinic of our Department; accordingly, with the aim of the study, we selected only non-smoker hypertensive patients, with stage ≤2 hypertension and with LVH, who were in monotherapy with olmesartan, 20 mg once a day. Fig 1 shows the selection flow of the final study population. The selection started from 388 (M/F = 243/145) consecutive outpatients referred for the first time to our clinic between October 2014 and May 2015 (newly diagnosed hypertensive outpatients); diagnosis of essential hypertension was considered as systolic blood pressure (SBP)≥140 mmHg and/or diastolic blood pressure (DBP)≥90 mmHg, in repeated home measurements, further confirmed by office measurement. Smokers were immediately excluded. Patients with office SBP≥180 mmHg and/or DBP≥110 mmHg, or with SBP <140 mmHg, were also excluded. Patients with a clinical history of CVD or alcohol consumption, with body mass index (BMI) ≥30, diabetes mellitus, low-density-lipoprotein-cholesterol levels (LDL-C)≥160 mg/dl, triglyceride levels (TG)≥200 mg/dl, albuminuria (diagnosed as the excretion of ≥30 mg/24 h of albumin), or with thyroid, liver or kidney diseases, were sequentially excluded from the analysis. Women taking hormone-based therapy were also excluded from the study. In accordance with our current clinical practice, secondary hypertension was systematically excluded, and complete clinical and laboratory examinations were performed and integrated with carotid echo-Doppler scan implemented by AS evaluation and with echocardiographic study. Clinical and instrumental examinations were completed within two weeks from first visit. Behavioral norms (caloric and salt intake restriction, weight loss, attitude to aerobic physical activity) were prescribed for at least six weeks. Blood samples were collected at time of diagnosis; no patients or controls were taking medications. BP was measured using a validated digital oscillometric device, Omron 705IT (HEM-759-E) (Omron Corporation-Healthcare, Kyoto, Japan). Three measurements performed with intervals of more than 2 minutes were then averaged. All analyses were performed on a venous blood sample taken at the medical center. Total cholesterol (TC), TG, high-density-lipoprotein-cholesterol (HDL-C), glucose and fibrinogen were measured by routine methods. LDL-C was calculated using the Friedewald formula. High-sensitivity C-reactive protein (HsCRP) was determined using an immunoturbidimetric latex assay kit. A blood sample was also obtained to count CD34+ cell number (FACSCalibur; Becton Dickinson and Co., Franklin Lakes, NJ, USA) and to evaluate miR expression (RealTime PCR); methods employed have been already explained in detail elsewhere[32].

When laboratory screening was completed, and after six weeks of non-pharmacological management, patients having SBP ≥140 mmHg and/or DBP ≥90 mmHg started drug therapy; patients who had received olmesartan as antihypertensive monotherapy (at usual dosage of 20 mg once a day)were then considered for the study; from clinical records, we analyzed data covering the 24weeks following the prescription (with office re-evaluation every 3–4 weeks).

In accordance with the study design, only patients with left ventricular hypertrophy (LVH), diagnosed as LV mass index (LVMI)≥102 g/m2 in men and ≥81 g/m2 in women[33,34], were finally included in the study (66 people). Finally, we identified 57 (M/F = 38/19) patients who had completed the needed observation period (24 weeks) without need of therapy modifications and who underwent the necessary clinical/instrumental examination.

Twenty-nine healthy subjects were also enrolled as control subjects from hospital personnel.

### Ethics statement

Written informed consent was obtained from all subjects in accordance with the Helsinki declaration; this observational study has been approved by the Ethics Committee of the University of Messina (prot. Number 07/15).

### Measurement of cIMT, arterial stiffness and LV parameters

Carotid ultrasonographic evaluation and AS assessment have already been described[32]. Briefly, semi-automated cIMT evaluation was performed using Aloka ProSound ALPHA10 with a 7–15 MHz linear array transducer; following ESC/ESH guidelines, we considered a mean cIMT ≥0.9 mm or plaque as carotid wall thickening. Augmentation index (AIx) and pulse wave velocity (PWV),like AS indices, were measured automatically by “eTRACKING” software. Following the method chosen to assess AS indices, we did not use pre-fixed cut-offs to classify normal or abnormal PWV and AIx. Since these indices are continuous variables, we considered PWV and AIx values compared to the normotensive control mean. LV examination was performed following American Society of Echocardiography recommendations, using a Vivid-7 ultrasound system (GE Medical System, Horten, Norway) equipped with a cardiac M4S transducer. LV mass was determined with the area-length method, and the LV mass index (LVMI) was calculated as LV mass/body surface area (BSA) (g/m2) ratio. LVH was diagnosed as a LV mass index (LVMI)≥102 g/m2 in men and ≥81 g/m2 in women[33,34].

### CD34+ cell identification and separation, enzyme and miR expression, ROS levels

Circulating CD34+ cells were identified and counted in peripheral blood by using flow cytometry (FACSCalibur; Becton Dickinson and Co., Franklin Lakes, NJ, USA), as already reported elsewhere [31]; for details please also see online supplement (S1 Text). Staining and analysis were performed in accordance with the International Society of Hematotherapy and Graft Engineering (ISHAGE) sequential strategy[35]. Gating strategies and sample analyses allowed the identification of the different phenotypes, by using the Macintosh CELLQuest software program (BD). Absolute CD34+ cell count was determined by comparing cellular events to bead events.

Molecular analyses were performed on CD34+ cells isolated from 15 ml of venous blood collected from each subject, after cell enrichment by using the MiniMACS system according to manufacturer’s instructions (Miltenyi Biotec Inc., CA, USA). Cell enrichment was validated by flow cytometry, confirming that at least 90% of separated cells were CD34+; for details please also see online supplement (S1 Text). The average values of miRs in samples from all control subjects were considered as the calibrator (1×sample). The results were expressed as an n-fold difference relative to the mean value (relative expression levels).

ROS generation in CD34+ cell-enriched samples was assessed using 2,7-dichlorofluorescin diacetate (DCFH-DA) by using a fluorimetric method[36]. ROS were expressed in fluorescence intensity relative units (FU). For further details please also see online supplement (S1 Text).

### Statistical methods

The Kolmogorov-Smirnov test was used to verify variables distribution. Since some variables had a non-normal distribution, and also given the relatively small size of our sample, we chose a non-parametric statistical approach. Consequently, to increase the power of statistics, we chose to use the non-parametric combination test (NPCT), based on a permutation solution within a resampling procedure, as already suggested elsewhere [37], to compare basal characteristics of hypertensive subjects with control subjects, and also in order to compare T1 vs T0 changes in hypertensive cases. Moreover, we performed comparisons between cases and controls also by the Anderson-Darling test. Accordingly, data are also shown as median ± standard deviation (SD). The mean difference of each variable at the two time-points was evaluated by the mean of the change of each patient, as mean relative Δ%, calculated as follows: (T1-T0)/T0*100. Interdependence analyses were performed by Spearman’s test. We performed a linear, stepwise, multivariate regression analysis to consider continuous and categorical variables together to assess the contribution of each variable to the study variables. A two-tailed alpha of 0.05 was used to denote statistical significance. To perform statistical analyses, we used the SPSS statistical package, version 17.0 (Chicago, IL, USA), and the NPC test 2.0 –Statistical software for multivariate permutation tests (Methodologica srl, Treviso, Italy).

## Results

Out of the 388 subjects referred to our Clinic over the reference period, we identified 57 patients with primary hypertension and left ventricular hypertrophy (LVH), selected as reported in **Fig 1**. Briefly, behavioral norms (caloric and salt intake restriction, weight loss, attitude to aerobic physical activity)were prescribed for at least six weeks, LVH was confirmed by transthoracic echocardiography, secondary hypertension was excluded, as were comorbidities, co-treatments, and target organ damage other than LVH. We selected only patients presenting with grade 1–2 hypertension (SBP 140–179 and/or DBP 90–109 mmHg) who started the treatment with olmesartan, 20 mg, who completed the observation time without need of therapy modifications, and who underwent a thorough clinical/instrumental examination.

**Table 1** summarizes the baseline characteristics of the study population. There were no differences regarding age, BMI, lipids and glucose between hypertensive subjects and controls. SBP and DBP values were higher in hypertensive subjects, as were also fibrinogen and CRP (both p<0.001). In addition, cIMT, AS indices and LVMI were higher in hypertensive cases (all p<0.001). With regard to cell estimation, we found that CD34+ cell number was higher in hypertensive subjects than in controls (3±1.1 vs 2.32±0.8 cells/μL, p = 0.006), but also intracellular ROS (81.8±33.9 vs 56.8±10.8, p<0.001), miR221 (1.29±0.5 vs 1±0.1, p<0.05) and miR222 (1.24±0.5 vs 1±0.3, p<0.05). Enrolled patients should complete the observation period without any change of therapy; accordingly, 9 patients dropped out of the study since they needed a second drug to reach the therapeutic goal.

**Table 1:** Characteristics of hypertensive patients at baseline (T0) and control subjects.

	Controls	Hypertensives (T0)	AD	NPC
**Number**	29	57		
**Gender (m/f)**	16/13	38/19		
	Mean±SD			p
**Age (years)**	39 (9)	40 (10)	0.356	0.808
**BMI (kg/m**^**2**^**)**	24.6 (3.1)	25 (4.1)	0.783	0.440
**SBP (mmHg)**	120 (20)	150 (10)	**<0.001**	**<0.001**
**DBP (mmHg)**	70 (15)	85 (10)	**<0.001**	**<0.001**
**TC (mg/dl)**	188 (38)	190 (58)	0.246	0.678
**HDL-C (mg/dl)**	49 (9.5)	47 (5.5)	0.133	0.242
**TG (mg/dl)**	112 (27)	101 (24)	0.216	0.303
**LDL-C (mg/dl)**	117.8 (36.4)	115 (57.3)	0.112	0.440
**Glucose (mg/dl)**	85 (9)	87 (17)	0.100	0.387
**HsCRP (mg/dl)**	0.39 (0.2)	0.9 (0.5)	**<0.001**	**<0.001**
**Fibrinogen (mg/dl)**	247 (89)	340 (112)	**<0.001**	**<0.001**
**AIx (%)**	-3.2 (5.7)	23.4 (22)	**<0.001**	**<0.001**
**PWV (m/s)**	4.9 (0.5)	8.2 (3.5)	**<0.001**	**<0.001**
**cIMT (mm)**	0.8 (0.3)	1.2 (0.5)	**<0.001**	**<0.001**
**LVMi**	91 (19.7)	125 (29)	**<0.001**	**<0.001**
**CD34+ cells (cells/μL)**	2.3 (1.6)	3.1 (1.9)	**0.011**	**0.006**
**ROS (FU)**	56.1 (21.5)	78.6 (61.6)	**0.002**	**<0.001**
**miR 221 (n-fold)**	0.98 (0.2)	1.26 (0.9)	**0.004**	**0.014**
**mir 222 (n-fold)**	0.94 (0.1)	1.23 (0.9)	**0.002**	**0.019**

Values are mean±SD. BMI: body mass index; SBP: systolic blood pressure; DBP: diastolic blood pressure; TC: total cholesterol; HDL-C: high density lipoprotein-cholesterol; TG: triglycerides; LDL-C: low density lipoprotein-cholesterol; HsCRP: high sensitivity C-reactive protein; AIx: augmentation index; PWV: pulse wave velocity; cIMT: carotid intima-media thickness; LVMi: left ventricular mass index; ROS: reactive oxygen species; miR: microRNA. p: statistical significance level for Anderson-Darling test (AD) test, or Non-Parametric Combination (NPC) test; hypertensives vs controls.

With olmesartan treatment (after 24 weeks),BP values were significantly reduced (SBP: Δ = -12.5% vs baseline, p<0.001; DBP:Δ = -16.6% vs baseline, p<0.001), as were fibrinogen (Δ = -7.9% vs baseline, p = 0.026), CRP (Δ = -16.4% vs baseline, p<0.001), AS indices (PWV: Δ = -33.0% vs baseline, p<0.001; AIx: Δ = -56.2% vs baseline, p<0.001), and also LVMi (Δ = -6.0% vs baseline, p<0.001). CD34+ cell number appeared to be increased (Δ = +17.2% vs baseline, p<0.001), while miRs 221/222 and ROS were reduced (all p<0.001 vs baseline). Comparisons between T0 and T1 are shown in **Table 2**.

**Table 2:** Characteristics of hypertensive patients at baseline (T0) and after 6-months antihypertensive therapy (T1).

	Hypertensives (T0)	Hypertensives (T1)		NPC
**Number**	57			
**Gender (m/f)**	38/19			
	Median (IQR)		Δ (%)	p
**BMI (kg/m**^**2**^**)**	25 (4.1)	25 (3.8)	-1.1	0.566
**SBP (mmHg)**	150(10)	135 (10)	-12.5	**<0.001**
**DBP (mmHg)**	85 (10)	75 (10)	-16.6	**<0.001**
**TC (mg/dl)**	190 (58)	180 (56)	-4.0	**<0.001**
**HDL-C (mg/dl)**	47 (5.5)	49.5 (10)	+2.67	0.437
**TG (mg/dl)**	101 (24)	100 (28)	-0.99	0.478
**LDL-C (mg/dl)**	115 (57.3)	112 (31)	-5.35	**<0.001**
**HsCRP (mg/dl)**	0.9 (0.5)	0.6 (0.3)	-29.7	**<0.001**
**Fibrinogen (mg/dl)**	340 (112)	308 (53)	-11.9	**0.026**
**AIx (%)**	23.4 (22)	11.2 (14.7)	-56.2	**<0.001**
**PWV (m/s)**	8.2 (3.5)	5.5 (2.3)	-33.0	**<0.001**
**LVMi**	125 (29)	118 (26.3)	-6.0	**<0.001**
**CD34+ cells (cells/μL)**	3.1 (1.9)	3.8 (1.5)	+17.2	**<0.001**
**ROS (FU)**	78.6 (61.6)	60 (40.6)	-16.9	**<0.001**
**miR 221 (n-fold)**	1.26 (0.9)	1.05 (0.95)	-23.6	**<0.001**
**miR 222 (n-fold)**	1.23 (0.9)	1.03 (1.01)	-16.0	**<0.001**

Values are mean±SD. BMI: body mass index; SBP: systolic blood pressure; DBP: diastolic blood pressure; TC: total cholesterol; HDL-C: high density lipoprotein-cholesterol; TG: triglycerides; LDL-C: low density lipoprotein-cholesterol; HsCRP: high sensitivity C-reactive protein; AIx: augmentation index; PWV: pulse wave velocity; cIMT: carotid intima-media thickness; LVMi: left ventricular mass index; ROS: reactive oxygen species; miR: microRNA. p: statistical significance level for Non-Parametric Combination (NPC) test for repeated measures test, T1 vs T0.

**Fig 2:**
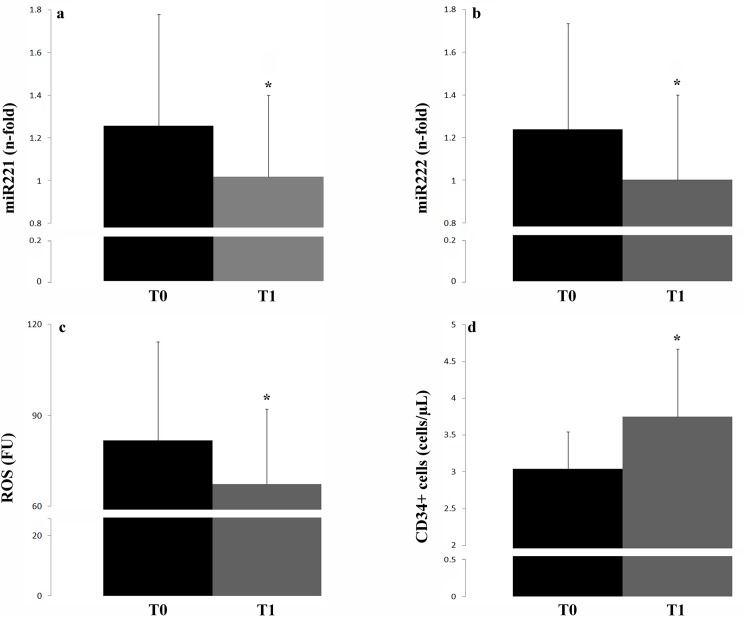
miR221 (a) and miR222 (b) expression (n-fold), ROS levels (c) and CD34+ cell count (d) at T0 and T1; *p<0.001 vs baseline. Significance level for Non-Parametric Combination (NPC) test.

**Fig 2** represents the main study variables at baseline and T1.

Interdependence analysis was performed (by Spearman’s test) on variable changes(delta); the main findings are shown in **Table 3**. BP value reduction correlated with fibrinogen (both SBP and DBP, p<0.005) and with PWV (SBP, p<0.05) reduction; moreover, we found that AIx changes were significantly correlated with miR221 reduction (rs = 0.321, p = 0.015), ROS reduction (rs = 0.271, p = 0.039) and LVMi reduction (rs = 0.346, p = 0.008). At T1, also lipid profile appeared to be improved (**Table 2**); LDL-C reduction appeared to be correlated with Δ LVMi (rs = 0.319, p = 0.015). ROS reduction was also correlated with miR221 reduced expression (rs = 0.332, p = 0.012), and fibrinogen reduction (rs = 0.289, p = 0.034).CD34+ cell count change at T1 appeared to be mainly correlated with fibrinogen reduction (rs = -0.625, p<0.001), and also with ROS reduction (rs = -0.341, p = 0.009).

**Table 3:** Correlations among variables.

	ΔSBP	ΔDBP	ΔFIB	ΔCRP	ΔCD34+	ΔmiR221	ΔmiR222	ΔAIx	ΔPWV	ΔLVMi
**ΔROS**	rs 0.180	rs 0.128	**rs 0.289**	rs -0.081	**rs -0.341**	**rs 0.332**	rs 0.057	**rs 0.271**	rs 0.031	rs 0.084
	p = 0.180	p = 0.341	**p = 0.034**	p = 0.550	**p = 0.009**	**p = 0.012**	p = 0.672	**p = 0.039**	p = 0.820	p = 0.533
**ΔCD34+**	rs -0.110	rs -0.165	**rs -0.625**	rs -0.046	—	rs 0.023	rs 0.064	rs 0.144	rs 0.050	rs 0.202
	p = 0.416	p = 0.221	**p = 0.001**	p = 0.736	—	p = 0.867	p = 0.638	p = 0.286	p = 0.711	p = 0.131
**ΔmiR221**	rs 0.166	rs 0.046	rs -0.174	**rs -0.268**	rs 0.023	—	rs 0.195	**rs 0.321**	rs 0.174	rs 0.039
	p = 0.217	p = 0.734	p = 0.195	**p = 0.044**	p = 0.868	—	p = 0.146	**p = 0.015**	p = 0.196	p = 0.773
**ΔmiR222**	rs 0.188	rs 0.033	rs -0.066	rs -0.087	rs -0.064	rs 0.195	—	rs 0.164	rs 0.053	rs 0.046
	p = 0.162	p = 0.808	p = 0.625	p = 0.519	p = 0.634	p = 0.146	—	p = 0.224	p = 0.693	p = 0.734

rs: correlation coefficient; p significance level for Spearman’s test.

Multiple regression analysis (**Table 4**) suggested ROS decrease (Δ ROS) as the main predictor for miR221 reduced expression, and also for miR222 decrease, while SBP decrease was associated with ROS decrease. CD34+ cell count increase appeared to be mainly attributable to fibrinogen decrease (Δ Fibrinogen).

**Table 4:** Multiple regression analysis.

Dependent variable	Predictors	Beta	T	P
**ΔCD34+ cells**	ΔFibrinogen	-0.586	-5.358	<0.001
**ΔmiR221**	ΔROS	0.699	7.254	<0.001
**ΔmiR222**	ΔROS	0.459	3.651	0.002
**ΔROS**	ΔSBP	0.360	2.858	0.006

Multiple regression analysis for Δ: CD34+ cell number, ROS and miRs in hypertensives; beta: standardized regression coefficient; T: t–test for beta; p: p-value for significance.

## Discussion

The present study provides evidence for the decrease of miR221/222 expression in CD34+CPCs from hypertensive patients with target organ damage, after a 6 month-treatment with olmesartan. Our data also confirm that olmesartan, in addition to lowering BP values, increases CD34+CPC number, and decreases ROS levels in CD34+CPCand plasma inflammatory markers. We also confirmed the improvement of LVMi and AS indices, as well as lipid profile, as already reported.

Most ATR1-mediated effects are triggered by AngII; ATR1 also may be activated independently from AngII[38], e.g. by mechanical stress and cellular stretching[39].ARBs are selective ATR1 antagonists available for clinical use in high BP treatment and are under examination for their potential protective effect against vascular inflammation and damaging, oxidative stress and CV remodelling. Some ARBs, including olmesartan, also work as inverse agonists[38], reducing the constitutive activity of ATR1and decreasing AngII- dependent and -independent mediated effects.

Activation ofATR1 induces signal transducers to phosphorylate the subunits of the NADPH oxidase enzymes, an important source of ROS[40].

ROS may act as signaling molecules for different protective functions, including endothelial repair and angiogenesis[16,41]. However, ROS accumulation induces oxidative stress and several harmful effects, including bioactive NO reduction, inflammation, cell senescence and apoptosis[42,43].Different factors, including AngII[44], shear stress[45] and miRs[26]may modulate NADPH oxidases; in particular, the sub-unit p47phox of NADPH oxidase complex was identified as afunctional target of miR221/222[16] suggesting that miR over-expression in progenitor cells may sustain oxidative stress.

In progenitor cells from healthy individuals, AngII was shown to accelerate senescence via NADPH oxidases and ROS production[46]. Moreover, progenitor cells isolated from hypertensive subjects showed a precocious cellular aging[47].Olmesartan has already been shown to reduce oxidative stress and endothelial inflammation[48,49]also by increasing anti-inflammatory and antioxidant molecules and NO availability[50]. Additionally, biological effects of molecules belonging to the “protective arm of RAS” appear to be enhanced by the use of ARBs, providing anti-inflammatory, anti-oxidant, anti-fibrotic and anti-apoptotic actions[51,52]. ATR1 and AT2R have recently been identified in progenitor cells, suggesting a role of AngII on these cells, mainly by interacting with ATR1, impairing antioxidative defense and inducing higher apoptosis [53]

It has been reported that hypertensive subjects may have a lower number of circulating EPCs[54,55,56]. We have already found a higher number of CD34+CPCs in hypertensive subjects as compared to normotensive controls, especially in hypertensive cases with arterial stiffening but not LVH. This discrepancy with other literature data may likely depend on the diversity of cellular phenotypes considered. Moreover, we suggest that the different amount of CD34+CPCs in hypertensive patients might also be related to a different degree of cell mobilization in the peripheral blood; other factors may also be involved, including disease status, e.g. the presence of early or more advanced lesions and target organ damage. However, this suggestion remains merely speculative since we have no mechanistic evidence in this regard.

Olmesartan has already been shown to increase EPC number in hypertensive subjects[50,57,58]. Although a role for AngII may be suggested, the pathway(s) through which ATR1 blockade may increase circulating progenitors currently remains unclear.

The present study confirms the increase in CD34+CPC number, and also shows a reduction in miR221/ 222 and ROS levels in CD34+CPCs. miR221/222 have been suggested to weaken cell proliferation and to induce apoptosis in human CD34+ cultured cells, particularly by targeting c-kit or indirectly regulating eNOS pathways[13,26]. Functionally, an miR may regulate the expression of multiple target genes; consistently, miR221/222 may trigger different pathways and molecules inducing cell dysfunction, such as transcription factors activating endothelial inflammatory molecules[27] or regulating endothelial mitochondrial energy metabolism[59].miR221/222-mediated pathways, including activation of p47phox of NADPH oxidase[16], result in enhanced oxidative stress, thus impairing cell function and survival[46,47]. In our hypertensive patients, the elevation of cells at T1 was mainly correlated with the lowering of fibrinogen and ROS; in addition, the reduction of ROS and, indirectly, of SBP, appeared to influence miR expression, suggesting that changes in BP or related mechanisms may play a role in normalizing the expression of intracellular miRs. Although miRs are thought to be involved in arterial remodelling[60] and atherogenesis[61], the impact of CV risk factors—including hypertension—on miR expression in CD34+CPCs remains to be clarified. Studies in animal models have shown that smooth muscle cells of rat express long non-coding RNAs, which seem to function as host transcripts for mirR221/222 and to be potentially regulated by AngII[62]. The modulation of miR221/222 expression is still unclear in human CPCs. SincemiR221/222expression is enhanced in CPCs from hypertensive subjects[31], and since miRs in microvascular ECs may modulate oxidative stress, via NADPH oxidase activation[16], and EPCs may express ATR1[53], one would assume that ARBs may be effective in reducing miR expression in CPCs. However, miR regulation may be targeted by different molecules through different pathways, including AngII and also ATR1-independent pathways. We aimed to provide an indirect estimate of the involvement of ATR1-mediated effects in modulatingmiR221/222expression in CPCsbyATR1 blockade with an ARB in humans.

Our hypertensive patients had increased levels of CRP and fibrinogen at baseline. Arterial hypertension, and related target organ damaging, is generally accompanied by a chronic inflammatory response[63], and several inflammatory molecules are thought to modulate cellular functions, including oxido-reductive balance. Plasma levels of CRP and fibrinogen were lowered after treatment, confirming the already reported anti-inflammatory effect of olmesartan[50,64].

Some limitations should be considered for this study. First, although our population was carefully studied and selected before data analysis, the sample size was relatively small. Second, our observation is limited to a short treatment time (24 weeks). Third, in this retrospective study our aim was to evaluate a homogeneous population of hypertensive cases in a monotherapy regimen. Since most of the selected patients were on treatment with olmesartan, we limited the observation to this population; thus, we do not know whether similar or different results could be reached by using a different antihypertensive drug/class. Last, ATR1 expression and signal transduction in CD34+ CPCs were not explored in this study; however, ATR1 and AngII–induced signaling and effects were already demonstrated in progenitor cells[53,65], but the potential link with miR expression and modulation was not previously investigated.

The observation that a treatment with olmesartan can modify miR221/222 expression in CD34+CPCs from LVH hypertensive subjects suggests a novel mechanism by which ATR1 blockade may increase the number of circulating progenitor cells. In addition to its well-known clinical effects, including blood pressure lowering, ATR1 blockade may improve endothelial homeostasis, providing multilevel CV protection in hypertensive subjects with target organ damage. Because the role of miR in progenitor cells is a new emerging area, additional investigations are needed to understand the pathways regulating the expression of miR221/222 in CPCs.

## Supporting information

S1 Text Methods; Measurement of carotid IMT and arterial stiffness, Echocardiographic Study, CD34+ cell identification and count, Molecular analysis, and Generation of ROS. (DOC)

## References

[1] Sidney LE, Branch MJ, Dunphy SE, Dua HS, Hopkinson A (2014) Concise review: evidence for CD34 as a common marker for diverse progenitors. Stem Cells 32: 1380–1389. 10.1002/stem.1661 24497003 PMC4260088

[2] de Boer HC, Hovens MM, van Oeveren-Rietdijk AM, Snoep JD, de Koning EJ, Tamsma JT, et al (2011) Human CD34+/KDR+ cells are generated from circulating CD34+ cells after immobilization on activated platelets. Arterioscler Thromb Vasc Biol 31: 408–415. 10.1161/ATVBAHA.110.216879 21030714

[3] Richardson MR, Yoder MC (2011) Endothelial progenitor cells: quo vadis? J Mol Cell Cardiol 50: 266–272. 10.1016/j.yjmcc.2010.07.009 20673769 PMC3444239

[4] Hirschi KK, Ingram DA, Yoder MC (2008) Assessing identity, phenotype, and fate of endothelial progenitor cells. Arterioscler Thromb Vasc Biol 28: 1584–1595. 10.1161/ATVBAHA.107.155960 18669889 PMC5244813

[5] Yoder MC (2013) Endothelial progenitor cell: a blood cell by many other names may serve similar functions. J Mol Med (Berl) 91: 285–295. 23371317 10.1007/s00109-013-1002-8 PMC3704045

[6] Fadini GP, Losordo D, Dimmeler S (2012) Critical re-evaluation of endothelial progenitor cell phenotypes for therapeutic and diagnostic use. Circulation Research 110: 624–637. 10.1161/CIRCRESAHA.111.243386 22343557 PMC3382070

[7] Chao H, Hirschi KK (2010) Hemato-vascular Origins of Endothelial Progenitor Cells? Microvascular research 79: 169–173. 10.1016/j.mvr.2010.02.003 20149806 PMC2857563

[8] Fadini GP, de Kreutzenberg SV, Coracina A, Baesso I, Agostini C, Tiengo A, et al (2006) Circulating CD34+ cells, metabolic syndrome, and cardiovascular risk. Eur Heart J 27: 2247–2255. 10.1093/eurheartj/ehl198 16912055

[9] Rigato M, Bittante C, Albiero M, Avogaro A, Fadini GP (2015) Circulating progenitor cell count predicts microvascular outcomes in type 2 diabetic patients. J Clin Endocrinol Metab: jc20151687. 10.1210/jc.2015-1687 25942480

[10] Bartel DP (2009) MicroRNAs: target recognition and regulatory functions. Cell 136: 215–233. 10.1016/j.cell.2009.01.002 19167326 PMC3794896

[11] Pillai RS, Bhattacharyya SN, Filipowicz W (2007) Repression of protein synthesis by miRNAs: how many mechanisms? Trends Cell Biol 17: 118–126. 10.1016/j.tcb.2006.12.007 17197185

[12] Ebert MS, Sharp PA (2012) Roles for microRNAs in conferring robustness to biological processes. Cell 149: 515–524. 10.1016/j.cell.2012.04.005 22541426 PMC3351105

[13] Felli N, Fontana L, Pelosi E, Botta R, Bonci D, Facchiano F, et al (2005) MicroRNAs 221 and 222 inhibit normal erythropoiesis and erythroleukemic cell growth via kit receptor down-modulation. Proc Natl Acad Sci U S A 102: 18081–18086. 10.1073/pnas.0506216102 16330772 PMC1312381

[14] Bonauer A, Boon RA, Dimmeler S (2010) Vascular microRNAs. Curr Drug Targets 11: 943–949. 20415654 10.2174/138945010791591313

[15] Poliseno L, Tuccoli A, Mariani L, Evangelista M, Citti L, Woods K, et al (2006) MicroRNAs modulate the angiogenic properties of HUVECs. Blood 108: 3068–3071. 10.1182/blood-2006-01-012369 16849646

[16] Shilo S, Roy S, Khanna S, Sen CK (2008) Evidence for the involvement of miRNA in redox regulated angiogenic response of human microvascular endothelial cells. Arterioscler Thromb Vasc Biol 28: 471–477. 10.1161/ATVBAHA.107.160655 18258815

[17] Urbich C, Kuehbacher A, Dimmeler S (2008) Role of microRNAs in vascular diseases, inflammation, and angiogenesis. Cardiovasc Res 79: 581–588. 10.1093/cvr/cvn156 18550634

[18] Albinsson S, Suarez Y, Skoura A, Offermanns S, Miano JM, Sessa WC (2010) MicroRNAs are necessary for vascular smooth muscle growth, differentiation, and function. Arterioscler Thromb Vasc Biol 30: 1118–1126. 10.1161/ATVBAHA.109.200873 20378849 PMC2880481

[19] Shi L, Liao J, Liu B, Zeng F, Zhang L (2015) Mechanisms and therapeutic potential of microRNAs in hypertension. Drug Discov Today 20: 1188–1204. 10.1016/j.drudis.2015.05.007 26004493 PMC4609581

[20] Morris BJ (2015) Renin, genes, microRNAs, and renal mechanisms involved in hypertension. Hypertension 65: 956–962. 10.1161/HYPERTENSIONAHA.114.04366 25601934

[21] Heggermont WA, Heymans S (2012) MicroRNAs are involved in end-organ damage during hypertension. Hypertension 60: 1088–1093. 10.1161/HYPERTENSIONAHA.111.187104 22987922

[22] Batkai S, Thum T (2012) MicroRNAs in hypertension: mechanisms and therapeutic targets. Curr Hypertens Rep 14: 79–87. 10.1007/s11906-011-0235-6 22052337

[23] Mendell JT, Olson EN (2012) MicroRNAs in stress signaling and human disease. Cell 148: 1172–1187. 10.1016/j.cell.2012.02.005 22424228 PMC3308137

[24] Quiat D, Olson EN (2013) MicroRNAs in cardiovascular disease: from pathogenesis to prevention and treatment. J Clin Invest 123: 11–18. 10.1172/JCI62876 23281405 PMC3533276

[25] Minami Y, Satoh M, Maesawa C, Takahashi Y, Tabuchi T, Itoh T, et al (2009) Effect of atorvastatin on microRNA 221 / 222 expression in endothelial progenitor cells obtained from patients with coronary artery disease. Eur J Clin Invest 39: 359–367. 10.1111/j.1365-2362.2009.02110.x 19371267

[26] Suarez Y, Fernandez-Hernando C, Pober JS, Sessa WC (2007) Dicer dependent microRNAs regulate gene expression and functions in human endothelial cells. Circ Res 100: 1164–1173. 10.1161/01.RES.0000265065.26744.17 17379831

[27] Zhu N, Zhang D, Chen S, Liu X, Lin L, Huang X, et al (2011) Endothelial enriched microRNAs regulate angiotensin II-induced endothelial inflammation and migration. Atherosclerosis 215: 286–293. 10.1016/j.atherosclerosis.2010.12.024 21310411

[28] Mandraffino G, Sardo MA, Riggio S, D'Ascola A, Alibrandi A, Saitta C, et al (2012) Circulating progenitor cells and the elderly: a seven-year observational study. Exp Gerontol 47: 394–400. 10.1016/j.exger.2012.03.007 22449458

[29] Mandraffino G, Sardo MA, Riggio S, D'Ascola A, Loddo S, Alibrandi A, et al (2010) Smoke exposure and circulating progenitor cells: evidence for modulation of antioxidant enzymes and cell count. Clin Biochem 43: 1436–1442. 10.1016/j.clinbiochem.2010.09.023 20888331

[30] Lo Gullo A, Mandraffino G, Bagnato G, Aragona CO, Imbalzano E, D'Ascola A, et al (2015) Vitamin D Status in Rheumatoid Arthritis: Inflammation, Arterial Stiffness and Circulating Progenitor Cell Number. PLoS One 10: e0134602 10.1371/journal.pone.0134602 26241902 PMC4524708

[31] Mandraffino G, Imbalzano E, Sardo MA, D'Ascola A, Mamone F, Lo Gullo A, et al (2014) Circulating progenitor cells in hypertensive patients with different degrees of cardiovascular involvement. J Hum Hypertens. 10.1038/jhh.2014.7 24553637

[32] Mandraffino G, Imbalzano E, Sardo MA, D'Ascola A, Mamone F, Lo Gullo A, et al (2014) Circulating progenitor cells in hypertensive patients with different degrees of cardiovascular involvement. J Hum Hypertens 28: 543–550. 10.1038/jhh.2014.7 24553637

[33] Lang RM, Bierig M, Devereux RB, Flachskampf FA, Foster E, Pellikka PA, et al (2005) Recommendations for chamber quantification: a report from the American Society of Echocardiography's Guidelines and Standards Committee and the Chamber Quantification Writing Group, developed in conjunction with the European Association of Echocardiography, a branch of the European Society of Cardiology. J Am Soc Echocardiogr 18: 1440–1463. 10.1016/j.echo.2005.10.005 16376782

[34] Chobanian AV, Bakris GL, Black HR, Cushman WC, Green LA, Izzo JL Jr., et al (2003) The Seventh Report of the Joint National Committee on Prevention, Detection, Evaluation, and Treatment of High Blood Pressure: the JNC 7 report. JAMA 289: 2560–2572. 10.1001/jama.289.19.2560 12748199

[35] Barnett D, Janossy G, Lubenko A, Matutes E, Newland A, Reilly JT (1999) Guideline for the flow cytometric enumeration of CD34+ haematopoietic stem cells. Prepared by the CD34+ haematopoietic stem cell working party. General Haematology Task Force of the British Committee for Standards in Haematology. Clin Lab Haematol 21: 301–308. 10646072 10.1046/j.1365-2257.1999.00253.x

[36] LeBel CP, Ischiropoulos H, Bondy SC (1992) Evaluation of the probe 2',7'-dichlorofluorescin as an indicator of reactive oxygen species formation and oxidative stress. Chem Res Toxicol 5: 227–231. 1322737 10.1021/tx00026a012

[37] Pesarin F, Salmaso L. (2010) Permutation Tests for Complex Data: Theory, Applications and Software: Wiley 1–11 p.

[38] Miura S, Fujino M, Hanzawa H, Kiya Y, Imaizumi S, Matsuo Y, et al (2006) Molecular mechanism underlying inverse agonist of angiotensin II type 1 receptor. J Biol Chem 281: 19288–19295. 10.1074/jbc.M602144200 16690611

[39] Yatabe J, Sanada H, Yatabe MS, Hashimoto S, Yoneda M, Felder RA, et al (2009) Angiotensin II type 1 receptor blocker attenuates the activation of ERK and NADPH oxidase by mechanical strain in mesangial cells in the absence of angiotensin II. Am J Physiol Renal Physiol 296: F1052–1060. 10.1152/ajprenal.00580.2007 19261744 PMC4067119

[40] Brandes RP, Kreuzer J (2005) Vascular NADPH oxidases: molecular mechanisms of activation. Cardiovasc Res 65: 16–27. 10.1016/j.cardiores.2004.08.007 15621030

[41] Ushio-Fukai M, Urao N (2009) Novel role of NADPH oxidase in angiogenesis and stem/progenitor cell function. Antioxid Redox Signal 11: 2517–2533. 10.1089/ARS.2009.2582 19309262 PMC2821135

[42] Touyz RM (2004) Reactive oxygen species, vascular oxidative stress, and redox signaling in hypertension: what is the clinical significance? Hypertension 44: 248–252. 10.1161/01.HYP.0000138070.47616.9d 15262903

[43] Paravicini TM, Touyz RM (2008) NADPH oxidases, reactive oxygen species, and hypertension: clinical implications and therapeutic possibilities. Diabetes Care 31 Suppl 2: S170–180. 18227481 10.2337/dc08-s247

[44] Mollnau H, Wendt M, Szocs K, Lassegue B, Schulz E, Oelze M, et al (2002) Effects of angiotensin II infusion on the expression and function of NAD(P)H oxidase and components of nitric oxide/cGMP signaling. Circ Res 90: E58–65. 11884382 10.1161/01.res.0000012569.55432.02

[45] Hwang J, Ing MH, Salazar A, Lassegue B, Griendling K, Navab M, et al (2003) Pulsatile versus oscillatory shear stress regulates NADPH oxidase subunit expression: implication for native LDL oxidation. Circ Res 93: 1225–1232. 10.1161/01.RES.0000104087.29395.66 14593003 PMC4433384

[46] Imanishi T, Hano T, Nishio I (2005) Angiotensin II accelerates endothelial progenitor cell senescence through induction of oxidative stress. J Hypertens 23: 97–104. 15643130 10.1097/00004872-200501000-00018

[47] Imanishi T, Moriwaki C, Hano T, Nishio I (2005) Endothelial progenitor cell senescence is accelerated in both experimental hypertensive rats and patients with essential hypertension. J Hypertens 23: 1831–1837. 16148606 10.1097/01.hjh.0000183524.73746.1b

[48] Tsuda M, Iwai M, Li JM, Li HS, Min LJ, Ide A, et al (2005) Inhibitory effects of AT1 receptor blocker, olmesartan, and estrogen on atherosclerosis via anti-oxidative stress. Hypertension 45: 545–551. 10.1161/01.HYP.0000157409.88971.fc 15723967

[49] Fliser D, Buchholz K, Haller H (2004) Antiinflammatory effects of angiotensin II subtype 1 receptor blockade in hypertensive patients with microinflammation. Circulation 110: 1103–1107. 10.1161/01.CIR.0000140265.21608.8E 15313950

[50] Calo LA, Dal Maso L, Pagnin E, Ravarotto V, Facco M, Boscaro E, et al (2014) Effect of olmesartan medoxomil on number and survival of circulating endothelial progenitor cells and calcitonin gene related peptide in hypertensive patients. J Hypertens 32: 193–199. 10.1097/HJH.0b013e32836522c3 24309489

[51] Singh KD, Karnik SS (2016) Angiotensin Receptors: Structure, Function, Signaling and Clinical Applications. J Cell Signal 1. 10.4172/jcs.1000111 PMC4976824 27512731

[52] Chappell MC (2016) Biochemical evaluation of the renin-angiotensin system: the good, bad, and absolute? Am J Physiol Heart Circ Physiol 310: H137–152. 10.1152/ajpheart.00618.2015 26475588 PMC4796631

[53] Endtmann C, Ebrahimian T, Czech T, Arfa O, Laufs U, Fritz M, et al (2011) Angiotensin II impairs endothelial progenitor cell number and function in vitro and in vivo: implications for vascular regeneration. Hypertension 58: 394–403. 10.1161/HYPERTENSIONAHA.110.169193 21825227

[54] Werner N, Nickenig G (2006) Influence of cardiovascular risk factors on endothelial progenitor cells: limitations for therapy? Arterioscler Thromb Vasc Biol 26: 257–266. 10.1161/01.ATV.0000198239.41189.5d 16322535

[55] Hill JM, Zalos G, Halcox JP, Schenke WH, Waclawiw MA, Quyyumi AA, et al (2003) Circulating endothelial progenitor cells, vascular function, and cardiovascular risk. N Engl J Med 348: 593–600. 10.1056/NEJMoa022287 12584367

[56] Pirro M, Schillaci G, Menecali C, Bagaglia F, Paltriccia R, Vaudo G, et al (2007) Reduced number of circulating endothelial progenitors and HOXA9 expression in CD34+ cells of hypertensive patients. J Hypertens 25: 2093–2099. 10.1097/HJH.0b013e32828e506d 17885552

[57] Bahlmann FH, de Groot K, Mueller O, Hertel B, Haller H, Fliser D (2005) Stimulation of endothelial progenitor cells: a new putative therapeutic effect of angiotensin II receptor antagonists. Hypertension 45: 526–529. 10.1161/01.HYP.0000159191.98140.89 15767470

[58] Gong X, Shao L, Fu YM, Zou Y (2015) Effects of olmesartan on endothelial progenitor cell mobilization and function in carotid atherosclerosis. Med Sci Monit 21: 1189–1193. 10.12659/MSM.892996 25913171 PMC4422112

[59] Xue Y, Wei Z, Ding H, Wang Q, Zhou Z, Zheng S, et al (2015) MicroRNA-19b/221/222 induces endothelial cell dysfunction via suppression of PGC-1alpha in the progression of atherosclerosis. Atherosclerosis 241: 671–681. 10.1016/j.atherosclerosis.2015.06.031 26117405

[60] Nazari-Jahantigh M, Wei Y, Schober A (2012) The role of microRNAs in arterial remodelling. Thromb Haemost 107: 611–618. 10.1160/TH11-12-0826 22371089

[61] Norata GD, Sala F, Catapano AL, Fernandez-Hernando C (2013) MicroRNAs and lipoproteins: a connection beyond atherosclerosis? Atherosclerosis 227: 209–215. 10.1016/j.atherosclerosis.2012.11.019 23260873 PMC4193445

[62] Leung A, Trac C, Jin W, Lanting L, Akbany A, Saetrom P, et al (2013) Novel long noncoding RNAs are regulated by angiotensin II in vascular smooth muscle cells. Circ Res 113: 266–278. 10.1161/CIRCRESAHA.112.300849 23697773 PMC3763837

[63] Bo S, Mandrile C, Milanesio N, Pagani A, Gentile L, Gambino R, et al (2012) Is left ventricular hypertrophy a low-level inflammatory state? A population-based cohort study. Nutr Metab Cardiovasc Dis 22: 668–676. 10.1016/j.numecd.2010.11.004 21429721

[64] Gutierrez-Fernandez M, Fuentes B, Rodriguez-Frutos B, Ramos-Cejudo J, Otero-Ortega L, Diez-Tejedor E (2015) Different protective and reparative effects of olmesartan in stroke according to time of administration and withdrawal. J Neurosci Res 93: 806–814. 10.1002/jnr.23532 25524827

[65] Roks AJM, Rodgers K, Walther T (2011) Effects of the renin angiotensin system on vasculogenesis-related progenitor cells. Current Opinion in Pharmacology 11: 162–174. 10.1016/j.coph.2011.01.002 21296616

