# Supplementary material for: Circulating progenitor cells in hypertensive subjects: Effectiveness of a treatment with olmesartan in improving cell number and miR profile in addition to expected pharmacological effects
Source: PLoS One. 2017 Mar 16;12(3):e0173030. doi: 10.1371/journal.pone.0173030 (PMC5354372; doi:10.1371/journal.pone.0173030)
Supplement: S1 Text — (DOC) [file pone.0173030.s001.doc]

**Circulating progenitor cells in hypertensive subjects: effectiveness of a treatment with olmesartan in improving cell number and miRs profile besides expected pharmacological effects.**

Giuseppe Mandraffino ^1*^, Caterina O. Aragona ^1^, Valentina Cairo ^1^, Michele Scuruchi ^2^, Angela D’Ascola ^2^, Angela Alibrandi ^3^, Saverio Loddo ^2^, Sebastiano Quartuccio ^1^, Carmela Morace ^1^, Enricomaria Mormina ^4^, Antonino Saitta ^1^, Egidio Imbalzano ^1^.

University of Messina

^1^ Department of Clinical and Experimental Medicine

^2^ Department of Biochemical, Physiological and Nutritional Sciences

^3^ Department of Statistics, University of Messina, Messina, Italy

^4^ Department of Biomedical Sciences and of Morphologic and Functional Images.

*Corresponding Author

E-mail: [gmandraffino@unime.it](mailto:gmandraffino@unime.it) (GM)

**SUPPLEMENTAL MATERIAL: Methods**

**Measurement of carotid IMT and arterial stiffness**

High-resolution B-mode ultrasound images (Vivid 7 Expert, GE Healthcare, Horten, Norway) with a 7-15 MHz linear array transducer were used to measure IMT. The carotid arteries were examined bilaterally in the areas of the common carotid (1cm proximal to the carotid bulb), the carotid bifurcation (1cm proximal to the flow divider) and the internal carotid artery (1cm distal to the flow divider). All measurements were determined manually on the far wall in longitudinal and transverse planes with anterior, lateral and posterior approaches. Two different readings were acquired for each projection. From B-mode images, single video frames were selected for IMT measurements. The IMT was defined as the distance between the lumen/intima and the media/adventitia interfaces. For each parameter the mean value was calculated. Two independent readers, who were blinded with respect to patients’ clinical and laboratory profile, made the measurements. The interobserver and intraobserver variability were evaluated on the measurements obtained from all subjects participating in the study. The interobserver variability of IMT measurements, as evaluated by comparing values obtained by two sets of scans evaluated by each reader, was 0.027mm (coefficient of variation 2.89%). The intraobserver variability was 0.021 mm (coefficient of variation 2.11%).

The parameters of vascular stiffness were automatically assessed at common carotid artery 2 cm before the bifurcation by Echo-tracking software (ALOKA Prosound alpha 10 ultrasound machine, 6-22-1, Mure, Mitaka-shi, Tokyo, Japan). Echo-tracking system implemented in the ultrasound machine allows accurate measurements of carotid diameter changes based on radio-frequencies (RF) signals, able to detect variations of the arterial diameters with a strictness of 0.01 mm. Pressure waveforms were noninvasively obtained using arterial diameter change waveforms calibrated based on blood pressure values (the software used needs to insert SBP and DBP values in the system of the machine for the calibration). On a 2D ultrasound image of the common carotid artery were positioned two sliders (tracking gates) at the front and back walls of the adventitia of the vessel. All acquisitions were synchronized with the electrocardiographic (ECG) signal. The software automatically calculates the main indices of arterial stiffness as a mean of five beats: β index, arterial compliance (AC), AIx, local PWV, Young elastic modulus (Ep), according to established formulas:

β-index = ln {(SBP/DBP)/[(Ds/Dd)/Dd]}; Ep = (SBP-DBP)/[(Ds-Dd)/Dd]; AC = π [(Ds x Ds)-(Dd x Dd)]/[4 (SBP-DBP)], where Dd is diastolic diameter and Ds systolic diameter. PWV is calculated with a one point method (15,16,29-31). The software calculates local PWV starting from the β-index through the report: PWV = √ (βp/2ρ), where p is the diastolic blood pressure and ρ is the density of blood (1050 kg/m3). AIx is calculated as the difference between the second (P2) and first (P1) systolic peaks (ΔP) of the central pressure waveform, as a percentage of the PP according to the formula AIx = P2-P1/PPx100 (expressed as a percentage). In young healthy subjects this value is usually negative.

**Echocardiographic Study**

Two-dimensional (2D) grayscale harmonic images were obtained in the left lateral position using a commercially available ultrasound system (Vivid-7GE Medical System, Horten, Norway) equipped with a cardiac M4S transducer. All measurements were averaged from three heart beats. LV wall thickness, diameters, volumes and EF were measured according to the American Society of echocardiography (ASE) recommendations. Global radial function was assessed by calculating both endocardial (e-FS) and mid-wall fractional shortening (m-FS). The end-diastolic relative wall thickness (RWT) was also calculated, and a cut-off value of ≥0.45 was taken into account to define a concentric remodeling. Circumferential end-systolic wall stress (c-ESS, 103 dynes/m2) was measured, according to the formula c-ESS = BP × R/2WT(1+WT/R), where R is the end-systolic radius, WT expresses the wall thickness and BP corresponds to the cuff systolic BP, as a surrogate of the intraventricular systolic pressure. The LV mass was determined with the area-length method, and the LV mass index was cal calculated as ratio LV mass/BSA (g/m2).The diagnosis of LV hypertrophy was based on a LV mass index (LVMI) >102 g/m2 in men and >81 g/m2 in women. PW Doppler mitral flow velocities (E and A), E/A ratio, E-wave deceleration time (Dt), left atrial (LA) areas and volumes were measured to assess LV diastolic function.

**CD34+ cells identification and count**

Flow cytometry (FACSCalibur; Becton Dickinson and Co., Franklin Lakes, NJ, USA) was used for cell identification. For this study, we identified and counted circulating CD34+ cells in peripheral blood. The cells were analyzed for the expression of surface antigens with direct multi-color analysis using fluorescein isothiocyanate (FITC)-conjugated, and phycoerythrin (PE)-conjugated monoclonal antibodies (mAbs) by flow cytometry analysis, as reported elsewhere . Staining and analysis were performed using the International Society of Hematotherapy and Graft Engineering (ISHAGE) sequential strategy . All peripheral blood samples were collected and stored in 0.34 M K3EDTA anticoagulant and analyzed within 2h. Fifty µL of peripheral blood was then incubated with 10 µL of FITC-conjugated anti-human CD45 mAb (Becton-Dickinson, BD, San Jose, CA, USA) and with 10 µL PE-conjugated anti-human CD34 mAb (BD), using the multiparameter flow cytometric lyse no-wash method PROCOUNT (BD) in a TRUCOUNT tube (BD) with a known number of fluorescent beads (as reported on label of each BD TRUCOUNT lot); BD Pharm Lyse was used as Lysing Buffer. Incubation was performed at room temperature for 15 min in the dark. Next, 7-amino-actinomycin D (7-AAD; VIA-PROBE, BD Pharmingen, San Diego, CA, USA) was added to identify dead cells. To avoid cell loss, no wash was performed. Flow cytometric acquisition and analysis were performed by FACSCalibur; the threshold was set on FITC fluorescence in a dot plot of CD45-FITC vs side scatter (SSC) to exclude debris and ensure that all leukocyte populations and microbeads were included. Gating strategies and sample analyses allowed the identification of CD34+ cells by using the Macintosh CELLQuest software program (BD). Cell number was expressed as absolute count following manufacturer instructions, as reported below; during analysis, the absolute number (cells/µL) of positive cells in the sample can be determined by comparing cellular events to beads events, according to the formula: cell population absolute count = (number of event in cell populations/number of events in absolute count bead region)*(number of bead-test/test volume). Only viable cells were counted; dead cells were excluded due to the use of 7-AAD. Consistenty, viable CD34+ cells were identified and counted.

**Molecular analysis**

Molecular analyses were performed on CD34+ cells; consequently, we collected from each subject 15 ml of venous blood. Mononuclear cells were separated from other blood components by density centrifugation with Lympholyte separation medium (Cedarlane, Burlington, ON, Canada), finally CD34+ population was enriched by magnetically cell sorting (Miltenyi Biotec Inc., CA, USA). Briefly, 15 ml of blood with heparin as anticoagulant were diluted with PBS (ratio 1:3) and carefully layered over 15ml of Lympholyte solution (Cedarlane) and finally centrifuged at 1400rpm for 30 min in a swinging-bucket rotor without break in order to obtain a well-defined lymphocyte monolayer in the interphase. Lymphocytes were then collected, transferred into a new centrifuge tube and washed twice with MACS buffer (Milteny Biotec). The pellet obtained were resuspended in a final volume of 300μl of MACS buffer and magnetically labeled adding 100μl of FcR blocking reagent (Milteny Biotec) and 100μl of CD34-conjugated MicroBeads (Milteny Biotec). After incubation for 30 min a 4 C° cells were washed with MACS buffer and processed through a MACS magnetic separation column (Miltenyi Biotec). The cell enrichment was validated by flow cytometry, confirming that at least 90% of separated cells were CD34+.

For RT-PCR of miR221 and mir222 total RNA was extracted from CD34+ population using the Total Purification Plus Kit (Norgen Biotek Corporation, Thorold, ON, Canada) and cDNA synthesis was obtained using the All-in-One miRNA first-strand cDNA synthesis kit (GeneCopoeia Inc., Rockville, MD, USA) according to the manufacturer’s recommendations. RT-PCR was performed using SYBR Premix DimerEraser (Clontech Laboratories, Inc., USA) through the SDS 7500 Real Time PCR instruments (Applied Biosystems, Foster City, CA). U6 snRNA was used as endogenous control and data were analyzed using the 2-ΔΔct method. The forward primer for miR-221 was 5’-GGCATGAACCTGGCATACA -3’ while the forward primer for miR222 was 5’-CTGCTGGAAGGTGTAGGTACC-3’. The reverse primers were the universal type primers provided by the kit All-in-One miRNA first-strand cDNA synthesis kit (GeneCopoeia Inc., Rockville, MD, USA). The primers for U6 snRNA were, respectively, 5’-CTCGCTTCGGCAGCACA-3’ and 5’-AACGCTTCACGAATTTGCGT-3’.

**Generation of ROS**

ROS generation in CD34+ cell-enriched samples was assessed using 2,7-dichlorofluorescin diacetate (DCFH-DA). ROS oxidizes DCFH-DA, generating a DCF fluorescent compound (2,7-dichlorofluorescein) which is used as an indirect marker of oxidant stress. DCF is detectable by flow cytometry at wavelengths of 488 and 525 nm, for excitation and emission, respectively. Immediately before the microplate reading, 10 μl of the fluorescent probe DCFH-DA, at a final concentration of 40 μM, was added to wells containing the cell suspension. ROS formation was monitored measuring 1x10^4^ cells per sample for 30min, with readings taken every 5 min. Data are expressed as fluorescence intensity relative units (FU).
